# (μ-1,4,7,10-Tetra­oxacyclo­dodeca­ne)bis­[(1,4,7,10-tetra­oxacyclo­dodeca­ne)lithium] bis­(perchlorate)

**DOI:** 10.1107/S1600536810009542

**Published:** 2010-03-24

**Authors:** Ilia A. Guzei, Lara C. Spencer, Lingyun Xiao, Ronald R. Burnette

**Affiliations:** a Department of Chemistry, University of Wisconsin-Madison, 1101 University Ave, Madison, WI 53706, USA; b Small Molecule Process & Product Development, AMGEN, One Amgen Center Drive, Thousand Oaks, CA 91320, USA; c School of Pharmacy, University of Wisconsin-Madison, 777 Highland Ave, Madison, WI 53705, USA

## Abstract

12-Crown-4 ether (12C4) and LiClO_4_ combine to form the ionic title compound, [Li_2_(C_8_H_16_O_4_)_3_](ClO_4_)_2_, which is com­posed of discrete Li/12C4 cations and perchlorate anions. In the [Li_2_(12C4)_3_]^2+^ cation there are two peripheral 12C4 ligands, which each form four Li—O bonds with only one Li^+^ atom. Additionally there is a central 12C4 in which diagonal O atoms form one Li—O bond each with both Li^+^ atoms. Therefore each Li^+^ atom is penta­coordinated in a distorted square-pyramidal geometry, forming four longer bonds to the O atoms on the peripheral 12C4 and one shorter bond to an O atom of the central 12C4. The cation occupies a crystallographic inversion centre located at the center of the ring of the central 12C4 ligand. The Li^+^ atom lies above the cavity of the peripheral 12C4 by 0.815 (2) Å because it is too large to fit in the cavity.

## Related literature

For applications of crown ethers, see: Jagannadh & Sarma (1999 ▶); Lehn (1973 ▶, 1995 ▶); Doyle & McCord (1998 ▶); Blasius *et al.* (1982 ▶); Blasius & Janzen (1982 ▶); Hayashita *et al.* (1992 ▶); Frühauf & Zeller (1991 ▶). For 12-crown-4 ether geometry, see: Raithby *et al.* (1997 ▶); Jones *et al.* (1997 ▶). For the size of the crown ether cavity and lithium ion, see: Shoham *et al.* (1983 ▶); Dalley (1978 ▶); Shannon (1976 ▶). For tris­(1,4,7,10-tetra­oxa­cyclo­dodeca­ne)dilithium bis­[tetra­hydrido­aluminate(III)], see: Bollmann & Olbrich (2004 ▶). Bond distances and angles were confirmed to be typical by a *Mogul* structural check (Bruno *et al.*, 2002 ▶). For a description of 1,4,7,10-tetra­oxacyclo­dodecane-trideuteroacetonitrile-lithium perchlorate, synthesized simultaneously with the title compound, see: Guzei *et al.* (2010 ▶). The outlier reflections were omitted based on the statistics test described by Prince & Nicholson (1983 ▶); Rollett (1988 ▶).
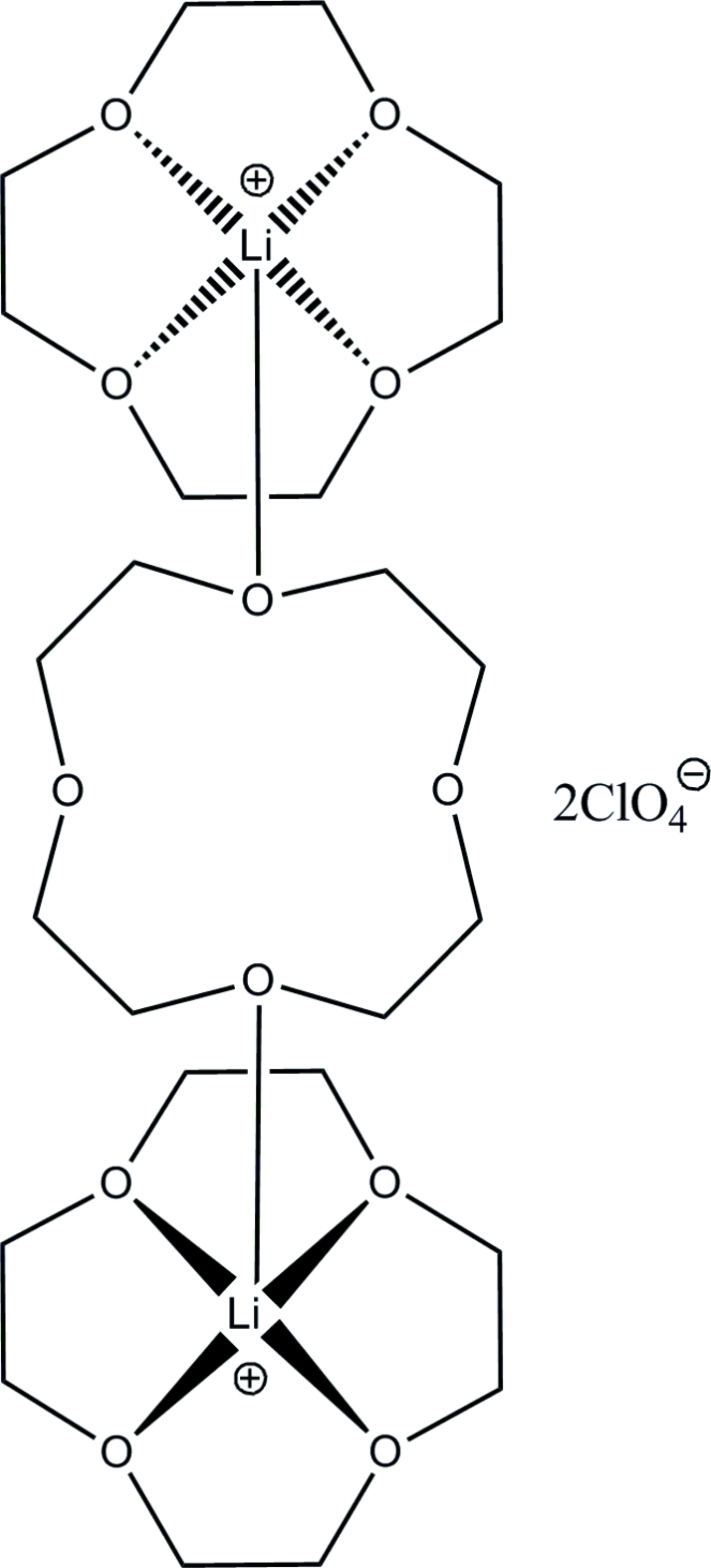


## Experimental

### 

#### Crystal data

[Li_2_(C_8_H_16_O_4_)_3_](ClO_4_)_2_
*M* *_r_* = 741.40
Monoclinic, 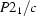

*a* = 7.7395 (7) Å
*b* = 14.1924 (13) Å
*c* = 15.2801 (14) Å
β = 95.962 (2)°
*V* = 1669.3 (3) Å^3^
*Z* = 2
Mo *K*α radiation
μ = 0.28 mm^−1^
*T* = 100 K
0.40 × 0.30 × 0.20 mm

#### Data collection

Bruker CCD-1000 area-detector diffractometer
Absorption correction: multi-scan (*SADABS*; Bruker, 2003 ▶) *T*_min_ = 0.897, *T*_max_ = 0.947
13593 measured reflections
3412 independent reflections
3139 reflections with *I* > 2σ(*I*)
*R*_int_ = 0.027

#### Refinement

*R*[*F* ^2^ > 2σ(*F* ^2^)] = 0.034
*wR*(*F* ^2^) = 0.092
*S* = 1.04
3412 reflections
217 parameters
H-atom parameters constrained
Δρ_max_ = 0.51 e Å^−3^
Δρ_min_ = −0.36 e Å^−3^

### 

Data collection: *SMART* (Bruker, 2003 ▶); cell refinement: *SAINT* (Bruker, 2003 ▶); data reduction: *SAINT*; program(s) used to solve structure: *SHELXTL* (Sheldrick, 2008 ▶); program(s) used to refine structure: *SHELXTL* and *FCF_filter* (Guzei, 2007 ▶); molecular graphics: *SHELXTL* and *DIAMOND* (Brandenburg, 1999 ▶); software used to prepare material for publication: *SHELXTL*, *publCIF* (Westrip, 2010 ▶) and *modiCIFer* (Guzei, 2007 ▶).

## Supplementary Material

Crystal structure: contains datablocks global, I. DOI: 10.1107/S1600536810009542/si2246sup1.cif

Structure factors: contains datablocks I. DOI: 10.1107/S1600536810009542/si2246Isup2.hkl

Additional supplementary materials:  crystallographic information; 3D view; checkCIF report

## Figures and Tables

**Table 1:** Selected bond lengths (Å)

O1—Li1	2.094 (3)
O2—Li1	2.079 (3)
O3—Li1	2.074 (3)
O4—Li1	2.061 (3)
O5—Li1	1.936 (3)

**Fig. 1.**
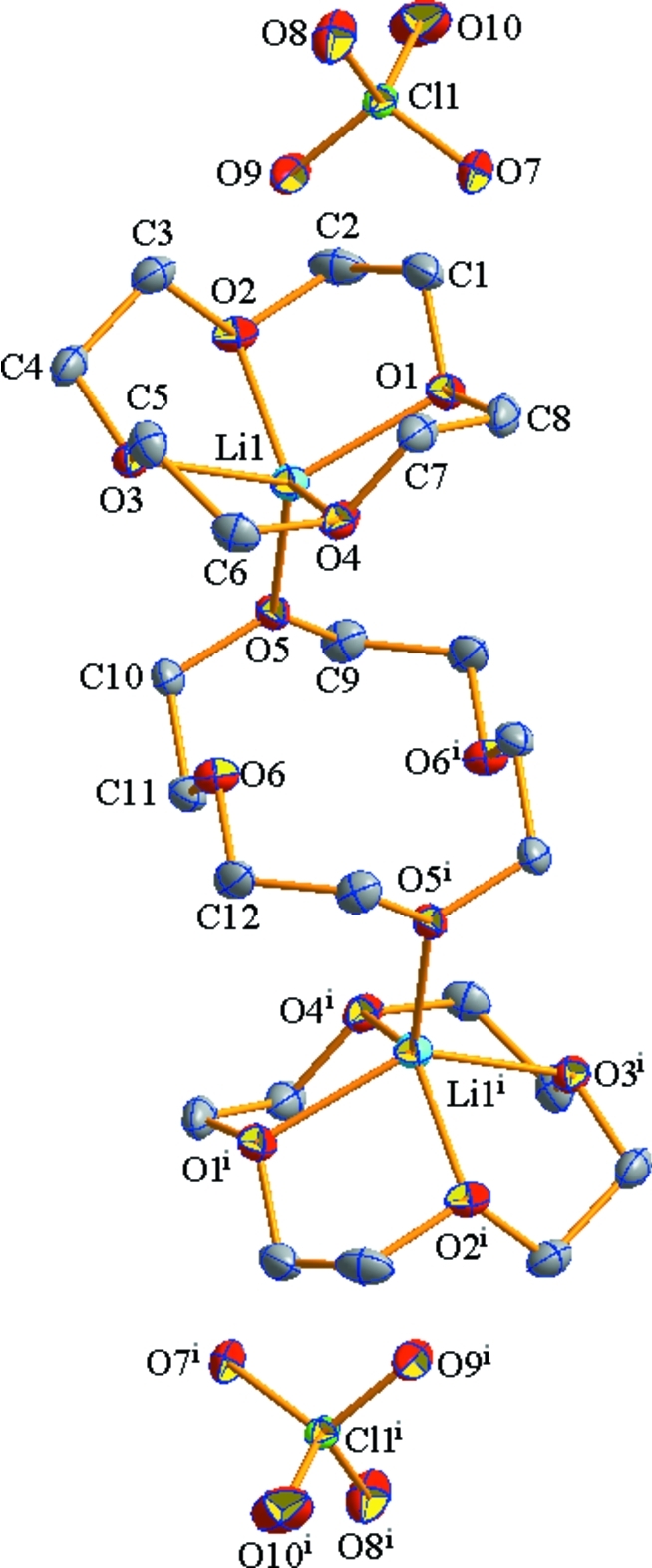
Molecular structure of (I). The thermal ellipsoids are shown at 50% probability level. All hydrogen atoms were omitted for clarity. Symmetry transformations used to generate equivalent atoms: i -x, -y, -z+2.

## supplementary crystallographic information

### Comment

Crown ethers complex with metal ions through the oxygen atoms with remarkable selectivity. They have high conformational flexibility, act as host molecules for various guests (Jagannadh *et al.*, 1999), and have a broad range of applications. Their importance has been studied in numerous fields such as molecular design (Lehn, 1973), supramolecular chemistry (Lehn, 1995), analytical chemistry (Doyle & McCord, 1998; Blasius *et al.*, 1982; Blasius & Janzen, 1982; Hayashita *et al.*, 1992) and medicine (Fruhauf & Zeller, 1991). In this study, the goal was to understand the nature of crown ether/Li^+^ complexes, and to extend its application to facilitate the characterization of host-guest type drug delivery systems. Thus, we are developing a systematic methodology based on experimental X-ray crystallography. As a result several novel complexes including the title compound (I) were synthesized.

12-crown-4 ether (12C4) and LiClO_4_ combine to form an ionic compound composed of discrete cations and anions. The cation is formed by two Li^+^ metals and three 12C4 ligands interacting to form a complex while the anion is uncomplexed perchlorate. In the cation, two of the 12C4 ligands are peripheral, each interacting with only one Li^+^. The third 12C4 lies between the two Li^+^ atoms and two opposite oxygen atoms each interact with one of the Li^+^ atoms. The cation occupies an inversion center located at the center of the ring of the central 12C4. Each Li^+^ is pentacoordinate with a distorted square pyramidal geometry forming four bonds to the oxygen atoms of a peripheral 12C4 and one bond to an oxygen atom belonging to the center 12C4. The Li—O bond to the central 12C4 is significantly shorter (1.936 (3) Å) than those to the oxygen atoms on the peripheral 12C4 (av. 2.077 (14) Å). The peripheral 12C4 has approximate C4 symmetry and is in the common [3333] conformation (Raithby *et al.*, 1997; Jones *et al.*, 1997) with the oxygen atoms being coplanar within of 0.013 Å . The central 12C4 is in the [66] conformation (Raithby *et al.*, 1997). The Li^+^ atom resides above the cavity of the peripheral 12C4 by 0.815 (2) Å. The average diagonal length measured between atom pairs O1/O3 and O2/O4 of the peripheral 12C4 is 3.8211 (15) Å resulting in an adjusted diameter of the cavity of 1.0211 Å (Shoham *et al.*, 1983; Dalley, 1978) . The Li^+^ has an ionic diameter between 1.18 Å and 1.52 Å; thus it is to large to fit in the cavity (Shannon, 1976).

The angles and distances involving the lithium atoms are similar to those in tris(1,4,7,10-tetraoxacyclododecane)-di-lithium bis(tetrahydridoaluminate(III)) (Bollmann & Olbrich, 2004) which contains the same cationic lithium complex as (I) with a different anion . A *Mogul* structural check confirmed that (I) exhibits typical geometrical parameters (Bruno *et al.*, 2002).

The Li^+^ cation complexes form sheets in the *ac* plane which stack along the *b* axis. The anions are positioned between adjacent sheets of cations.

### Experimental

All chemicals were purchased from the Aldrich Chemical Co. Inc. and were used as received. 12-crown-4 (12C4, C_8_H_16_O_4_, 98% pure) and lithium perchlorate (LiClO_4_, 99% pure) were separately dissolved in acetonitrile-d_3_ (CD_3_CN, 99% pure). These two solutions were then mixed together according to a 1:1 molar ratio of 12C4/LiClO_4_.The final solution was kept in a desiccator and the solvent was allowed to evaporate gradually in order to produce a supersaturated solution. The supersaturated solution was stored at –20 °C refrigerator, until crystals formed after 48 hours. Two types of colorless crystals suitable for X-ray diffraction were obtained and separated from the solution, one of which was compound (I) the other being 1,4,7,10-tetraoxacyclododecane-trideuteroacetonitrile-lithium perchlorate (Guzei *et al.*, 2010).

### Refinement

All H atoms were placed in idealized locations and refined as riding, with C—H=0.99 Å and *U*_iso_(H) = 1.2*U*_eq_(C).

The outlier reflections were omitted based on the statistics test described in Prince, E. and Nicholson, W. L. (1983) Acta Cryst. A39, 407-410 and Rollett J. S. (1988) Crystallographic Computing 4, 149-166. Oxford University Press, and implemented in program FCF_filter (Guzei, 2007). The number of omitted outliers is 2..

### Crystal data

**Table:**

[Li_2_(C_8_H_16_O_4_)_3_](ClO_4_)_2_	*F*(000) = 784
*M**_r_* = 741.40	*D*_x_ = 1.475 Mg m^−^^3^
Monoclinic, *P*2_1_/*c*	Mo *K*α radiation, λ = 0.71073 Å
Hall symbol: -P 2ybc	Cell parameters from 999 reflections
*a* = 7.7395 (7) Å	θ = 2.7–26.4°
*b* = 14.1924 (13) Å	µ = 0.28 mm^−^^1^
*c* = 15.2801 (14) Å	*T* = 100 K
β = 95.962 (2)°	Block, colourless
*V* = 1669.3 (3) Å^3^	0.40 × 0.30 × 0.20 mm
*Z* = 2	

### Data collection

**Table:**

Bruker CCD-1000 area-detector diffractometer	3412 independent reflections
Radiation source: fine-focus sealed tube	3139 reflections with *I* > 2σ(*I*)
graphite	*R*_int_ = 0.027
0.30° ω and 0.4 ° φ scans	θ_max_ = 26.4°, θ_min_ = 2.7°
Absorption correction: multi-scan (*SADABS*; Bruker, 2003)	*h* = −9→9
*T*_min_ = 0.897, *T*_max_ = 0.947	*k* = −17→17
13593 measured reflections	*l* = −19→19

### Refinement

**Table:**

Refinement on *F*^2^	Primary atom site location: structure-invariant direct methods
Least-squares matrix: full	Secondary atom site location: difference Fourier map
*R*[*F*^2^ > 2σ(*F*^2^)] = 0.034	Hydrogen site location: inferred from neighbouring sites
*wR*(*F*^2^) = 0.092	H-atom parameters constrained
*S* = 1.04	*w* = 1/[σ^2^(*F*_o_^2^) + (0.0467*P*)^2^ + 1.027*P*] where *P* = (*F*_o_^2^ + 2*F*_c_^2^)/3
3412 reflections	(Δ/σ)_max_ < 0.001
217 parameters	Δρ_max_ = 0.51 e Å^−^^3^
0 restraints	Δρ_min_ = −0.36 e Å^−^^3^

### Special details

**Table:**

Geometry. All esds (except the esd in the dihedral angle between two l.s. planes) are estimated using the full covariance matrix. The cell esds are taken into account individually in the estimation of esds in distances, angles and torsion angles; correlations between esds in cell parameters are only used when they are defined by crystal symmetry. An approximate (isotropic) treatment of cell esds is used for estimating esds involving l.s. planes.
Refinement. Refinement of *F*^2^ against ALL reflections. The weighted *R*-factor wR and goodness of fit *S* are based on *F*^2^, conventional *R*-factors *R* are based on *F*, with *F* set to zero for negative *F*^2^. The threshold expression of *F*^2^ > σ(*F*^2^) is used only for calculating *R*-factors(gt) etc. and is not relevant to the choice of reflections for refinement. *R*-factors based on *F*^2^ are statistically about twice as large as those based on *F*, and *R*- factors based on ALL data will be even larger.

### Fractional atomic coordinates and isotropic or equivalent isotropic displacement parameters (Å^2^)

**Table:**

	*x*	*y*	*z*	*U*_iso_*/*U*_eq_	
O1	0.37224 (13)	−0.00939 (8)	0.79331 (7)	0.0253 (2)	
O2	0.14456 (14)	−0.11875 (7)	0.68986 (7)	0.0228 (2)	
O3	−0.10708 (13)	0.00835 (7)	0.70392 (6)	0.0206 (2)	
O4	0.11574 (14)	0.11599 (7)	0.80927 (7)	0.0223 (2)	
O5	0.01430 (12)	−0.10039 (7)	0.88536 (6)	0.0188 (2)	
O6	−0.20229 (14)	0.05033 (7)	0.94017 (7)	0.0233 (2)	
Li1	0.1036 (3)	−0.02805 (17)	0.79255 (15)	0.0201 (5)	
C1	0.4243 (2)	−0.04051 (12)	0.71080 (10)	0.0279 (3)	
H1A	0.5516	−0.0498	0.7151	0.033*	
H1B	0.3909	0.0063	0.6641	0.033*	
C2	0.3315 (2)	−0.13188 (12)	0.69088 (11)	0.0303 (4)	
H2A	0.3585	−0.1557	0.6329	0.036*	
H2B	0.3719	−0.1792	0.7360	0.036*	
C3	0.0620 (2)	−0.08202 (11)	0.60923 (10)	0.0261 (3)	
H3A	0.0564	−0.1304	0.5624	0.031*	
H3B	0.1261	−0.0267	0.5900	0.031*	
C4	−0.1181 (2)	−0.05422 (12)	0.62882 (10)	0.0261 (3)	
H4A	−0.1794	−0.0223	0.5770	0.031*	
H4B	−0.1849	−0.1112	0.6414	0.031*	
C5	−0.0971 (2)	0.10586 (11)	0.68153 (10)	0.0243 (3)	
H5A	−0.2113	0.1291	0.6550	0.029*	
H5B	−0.0103	0.1158	0.6392	0.029*	
C6	−0.0433 (2)	0.15582 (11)	0.76647 (10)	0.0255 (3)	
H6A	−0.0256	0.2236	0.7548	0.031*	
H6B	−0.1366	0.1503	0.8058	0.031*	
C7	0.2697 (2)	0.15160 (11)	0.77685 (10)	0.0251 (3)	
H7A	0.2919	0.2173	0.7966	0.030*	
H7B	0.2586	0.1501	0.7117	0.030*	
C8	0.4132 (2)	0.08828 (12)	0.81403 (10)	0.0277 (3)	
H8A	0.5222	0.1058	0.7895	0.033*	
H8B	0.4315	0.0963	0.8787	0.033*	
C9	0.1074 (2)	−0.16375 (11)	0.94693 (10)	0.0236 (3)	
H9A	0.1499	−0.2182	0.9148	0.028*	
H9B	0.0280	−0.1878	0.9886	0.028*	
C10	−0.17131 (18)	−0.10405 (11)	0.88613 (9)	0.0211 (3)	
H10A	−0.2075	−0.1703	0.8930	0.025*	
H10B	−0.2275	−0.0808	0.8290	0.025*	
C11	−0.23197 (18)	−0.04594 (11)	0.95912 (10)	0.0219 (3)	
H11A	−0.3572	−0.0569	0.9632	0.026*	
H11B	−0.1670	−0.0636	1.0160	0.026*	
C12	−0.2582 (2)	0.11475 (11)	1.00296 (10)	0.0248 (3)	
H12A	−0.3248	0.0802	1.0447	0.030*	
H12B	−0.3363	0.1622	0.9723	0.030*	
Cl1	0.42590 (4)	0.18127 (2)	0.53628 (2)	0.01772 (11)	
O7	0.49851 (14)	0.23355 (8)	0.61216 (7)	0.0266 (3)	
O8	0.38979 (16)	0.24318 (8)	0.46246 (8)	0.0325 (3)	
O9	0.26490 (16)	0.13825 (9)	0.55554 (7)	0.0334 (3)	
O10	0.54620 (18)	0.11021 (10)	0.51571 (9)	0.0421 (3)	

### Atomic displacement parameters (Å^2^)

**Table:**

	*U*^11^	*U*^22^	*U*^33^	*U*^12^	*U*^13^	*U*^23^
O1	0.0200 (5)	0.0360 (6)	0.0202 (5)	−0.0012 (4)	0.0035 (4)	0.0050 (4)
O2	0.0284 (6)	0.0210 (5)	0.0197 (5)	−0.0002 (4)	0.0064 (4)	0.0007 (4)
O3	0.0213 (5)	0.0225 (5)	0.0179 (5)	−0.0017 (4)	0.0012 (4)	0.0025 (4)
O4	0.0266 (5)	0.0227 (5)	0.0177 (5)	−0.0050 (4)	0.0026 (4)	−0.0001 (4)
O5	0.0162 (5)	0.0221 (5)	0.0183 (5)	0.0005 (4)	0.0026 (4)	0.0035 (4)
O6	0.0276 (5)	0.0241 (5)	0.0191 (5)	0.0013 (4)	0.0060 (4)	−0.0004 (4)
Li1	0.0201 (11)	0.0215 (12)	0.0188 (11)	−0.0005 (9)	0.0028 (9)	0.0021 (9)
C1	0.0204 (7)	0.0401 (9)	0.0240 (8)	0.0036 (6)	0.0065 (6)	0.0031 (7)
C2	0.0344 (9)	0.0293 (8)	0.0295 (8)	0.0115 (7)	0.0138 (7)	0.0026 (7)
C3	0.0332 (8)	0.0269 (8)	0.0181 (7)	−0.0032 (6)	0.0033 (6)	−0.0031 (6)
C4	0.0287 (8)	0.0304 (8)	0.0183 (7)	−0.0091 (6)	−0.0015 (6)	−0.0031 (6)
C5	0.0217 (7)	0.0234 (7)	0.0273 (8)	0.0013 (6)	0.0001 (6)	0.0063 (6)
C6	0.0266 (8)	0.0219 (7)	0.0290 (8)	0.0054 (6)	0.0075 (6)	0.0020 (6)
C7	0.0283 (8)	0.0264 (8)	0.0211 (7)	−0.0099 (6)	0.0043 (6)	−0.0009 (6)
C8	0.0229 (7)	0.0377 (9)	0.0220 (7)	−0.0124 (6)	−0.0006 (6)	−0.0002 (6)
C9	0.0288 (8)	0.0193 (7)	0.0226 (7)	0.0045 (6)	0.0020 (6)	0.0022 (6)
C10	0.0167 (7)	0.0260 (7)	0.0207 (7)	−0.0057 (5)	0.0019 (5)	−0.0007 (6)
C11	0.0186 (7)	0.0269 (8)	0.0206 (7)	−0.0004 (6)	0.0050 (5)	0.0031 (6)
C12	0.0228 (7)	0.0292 (8)	0.0223 (7)	0.0086 (6)	0.0012 (6)	−0.0023 (6)
Cl1	0.01952 (18)	0.01895 (18)	0.01472 (18)	−0.00176 (12)	0.00186 (12)	0.00107 (11)
O7	0.0229 (5)	0.0326 (6)	0.0233 (5)	−0.0044 (4)	−0.0018 (4)	−0.0071 (5)
O8	0.0369 (6)	0.0321 (6)	0.0267 (6)	−0.0082 (5)	−0.0056 (5)	0.0132 (5)
O9	0.0339 (6)	0.0468 (7)	0.0197 (5)	−0.0226 (5)	0.0043 (5)	−0.0008 (5)
O10	0.0496 (8)	0.0424 (8)	0.0346 (7)	0.0217 (6)	0.0055 (6)	−0.0075 (6)

### Geometric parameters (Å, °)

**Table:**

O1—C1	1.4326 (18)	C4—H4B	0.9900
O1—C8	1.450 (2)	C5—C6	1.499 (2)
O1—Li1	2.094 (3)	C5—H5A	0.9900
O2—C3	1.4264 (18)	C5—H5B	0.9900
O2—C2	1.4573 (19)	C6—H6A	0.9900
O2—Li1	2.079 (3)	C6—H6B	0.9900
O3—C5	1.4296 (18)	C7—C8	1.494 (2)
O3—C4	1.4466 (18)	C7—H7A	0.9900
O3—Li1	2.074 (3)	C7—H7B	0.9900
O4—C7	1.4295 (18)	C8—H8A	0.9900
O4—C6	1.4473 (18)	C8—H8B	0.9900
O4—Li1	2.061 (3)	C9—C12^i^	1.499 (2)
O5—C10	1.4388 (16)	C9—H9A	0.9900
O5—C9	1.4394 (17)	C9—H9B	0.9900
O5—Li1	1.936 (3)	C10—C11	1.501 (2)
O6—C11	1.4203 (18)	C10—H10A	0.9900
O6—C12	1.4247 (18)	C10—H10B	0.9900
C1—C2	1.498 (2)	C11—H11A	0.9900
C1—H1A	0.9900	C11—H11B	0.9900
C1—H1B	0.9900	C12—C9^i^	1.499 (2)
C2—H2A	0.9900	C12—H12A	0.9900
C2—H2B	0.9900	C12—H12B	0.9900
C3—C4	1.508 (2)	Cl1—O10	1.4294 (12)
C3—H3A	0.9900	Cl1—O8	1.4343 (11)
C3—H3B	0.9900	Cl1—O7	1.4407 (11)
C4—H4A	0.9900	Cl1—O9	1.4451 (11)
			
C1—O1—C8	114.32 (12)	C6—C5—H5A	110.6
C1—O1—Li1	109.00 (11)	O3—C5—H5B	110.6
C8—O1—Li1	108.46 (11)	C6—C5—H5B	110.6
C3—O2—C2	114.29 (11)	H5A—C5—H5B	108.8
C3—O2—Li1	109.63 (11)	O4—C6—C5	110.67 (12)
C2—O2—Li1	107.45 (11)	O4—C6—H6A	109.5
C5—O3—C4	113.86 (11)	C5—C6—H6A	109.5
C5—O3—Li1	109.81 (11)	O4—C6—H6B	109.5
C4—O3—Li1	110.02 (11)	C5—C6—H6B	109.5
C7—O4—C6	113.93 (11)	H6A—C6—H6B	108.1
C7—O4—Li1	109.66 (11)	O4—C7—C8	105.61 (12)
C6—O4—Li1	107.81 (11)	O4—C7—H7A	110.6
C10—O5—C9	113.85 (11)	C8—C7—H7A	110.6
C10—O5—Li1	117.26 (11)	O4—C7—H7B	110.6
C9—O5—Li1	128.14 (11)	C8—C7—H7B	110.6
C11—O6—C12	114.41 (11)	H7A—C7—H7B	108.7
O5—Li1—O4	116.76 (12)	O1—C8—C7	110.79 (12)
O5—Li1—O3	107.10 (12)	O1—C8—H8A	109.5
O4—Li1—O3	81.74 (10)	C7—C8—H8A	109.5
O5—Li1—O2	108.58 (12)	O1—C8—H8B	109.5
O4—Li1—O2	134.35 (13)	C7—C8—H8B	109.5
O3—Li1—O2	80.36 (9)	H8A—C8—H8B	108.1
O5—Li1—O1	119.53 (12)	O5—C9—C12^i^	110.75 (12)
O4—Li1—O1	80.89 (10)	O5—C9—H9A	109.5
O3—Li1—O1	133.22 (12)	C12^i^—C9—H9A	109.5
O2—Li1—O1	81.58 (10)	O5—C9—H9B	109.5
O1—C1—C2	105.85 (12)	C12^i^—C9—H9B	109.5
O1—C1—H1A	110.6	H9A—C9—H9B	108.1
C2—C1—H1A	110.6	O5—C10—C11	112.07 (11)
O1—C1—H1B	110.6	O5—C10—H10A	109.2
C2—C1—H1B	110.6	C11—C10—H10A	109.2
H1A—C1—H1B	108.7	O5—C10—H10B	109.2
O2—C2—C1	110.23 (12)	C11—C10—H10B	109.2
O2—C2—H2A	109.6	H10A—C10—H10B	107.9
C1—C2—H2A	109.6	O6—C11—C10	107.90 (11)
O2—C2—H2B	109.6	O6—C11—H11A	110.1
C1—C2—H2B	109.6	C10—C11—H11A	110.1
H2A—C2—H2B	108.1	O6—C11—H11B	110.1
O2—C3—C4	105.34 (12)	C10—C11—H11B	110.1
O2—C3—H3A	110.7	H11A—C11—H11B	108.4
C4—C3—H3A	110.7	O6—C12—C9^i^	111.52 (12)
O2—C3—H3B	110.7	O6—C12—H12A	109.3
C4—C3—H3B	110.7	C9^i^—C12—H12A	109.3
H3A—C3—H3B	108.8	O6—C12—H12B	109.3
O3—C4—C3	109.81 (12)	C9^i^—C12—H12B	109.3
O3—C4—H4A	109.7	H12A—C12—H12B	108.0
C3—C4—H4A	109.7	O10—Cl1—O8	109.72 (8)
O3—C4—H4B	109.7	O10—Cl1—O7	109.37 (7)
C3—C4—H4B	109.7	O8—Cl1—O7	110.20 (7)
H4A—C4—H4B	108.2	O10—Cl1—O9	109.99 (9)
O3—C5—C6	105.53 (12)	O8—Cl1—O9	108.50 (7)
O3—C5—H5A	110.6	O7—Cl1—O9	109.04 (7)
			
C10—O5—Li1—O4	79.93 (16)	C1—O1—Li1—O4	−119.04 (11)
C9—O5—Li1—O4	−110.63 (15)	C8—O1—Li1—O4	6.01 (12)
C10—O5—Li1—O3	−9.32 (17)	C1—O1—Li1—O3	−49.5 (2)
C9—O5—Li1—O3	160.13 (11)	C8—O1—Li1—O3	75.50 (19)
C10—O5—Li1—O2	−94.65 (14)	C1—O1—Li1—O2	18.65 (12)
C9—O5—Li1—O2	74.80 (17)	C8—O1—Li1—O2	143.69 (10)
C10—O5—Li1—O1	174.62 (12)	C8—O1—C1—C2	−165.58 (12)
C9—O5—Li1—O1	−15.9 (2)	Li1—O1—C1—C2	−44.04 (15)
C7—O4—Li1—O5	142.15 (13)	C3—O2—C2—C1	81.88 (16)
C6—O4—Li1—O5	−93.29 (15)	Li1—O2—C2—C1	−40.01 (16)
C7—O4—Li1—O3	−112.81 (10)	O1—C1—C2—O2	56.83 (16)
C6—O4—Li1—O3	11.75 (11)	C2—O2—C3—C4	−167.89 (12)
C7—O4—Li1—O2	−45.1 (2)	Li1—O2—C3—C4	−47.21 (15)
C6—O4—Li1—O2	79.51 (19)	C5—O3—C4—C3	89.63 (15)
C7—O4—Li1—O1	23.59 (12)	Li1—O3—C4—C3	−34.13 (15)
C6—O4—Li1—O1	148.15 (10)	O2—C3—C4—O3	53.90 (15)
C5—O3—Li1—O5	133.72 (12)	C4—O3—C5—C6	−166.51 (12)
C4—O3—Li1—O5	−100.20 (13)	Li1—O3—C5—C6	−42.64 (14)
C5—O3—Li1—O4	18.16 (12)	C7—O4—C6—C5	82.64 (15)
C4—O3—Li1—O4	144.25 (10)	Li1—O4—C6—C5	−39.31 (15)
C5—O3—Li1—O2	−119.67 (11)	O3—C5—C6—O4	55.08 (15)
C4—O3—Li1—O2	6.42 (12)	C6—O4—C7—C8	−168.01 (12)
C5—O3—Li1—O1	−51.0 (2)	Li1—O4—C7—C8	−47.09 (14)
C4—O3—Li1—O1	75.10 (19)	C1—O1—C8—C7	88.01 (15)
C3—O2—Li1—O5	128.64 (12)	Li1—O1—C8—C7	−33.84 (15)
C2—O2—Li1—O5	−106.61 (13)	O4—C7—C8—O1	54.00 (15)
C3—O2—Li1—O4	−44.6 (2)	C10—O5—C9—C12^i^	−134.05 (13)
C2—O2—Li1—O4	80.17 (19)	Li1—O5—C9—C12^i^	56.20 (18)
C3—O2—Li1—O3	23.72 (12)	C9—O5—C10—C11	81.83 (15)
C2—O2—Li1—O3	148.47 (10)	Li1—O5—C10—C11	−107.23 (14)
C3—O2—Li1—O1	−112.94 (11)	C12—O6—C11—C10	178.36 (11)
C2—O2—Li1—O1	11.80 (12)	O5—C10—C11—O6	67.70 (15)
C1—O1—Li1—O5	125.29 (14)	C11—O6—C12—C9^i^	112.52 (14)
C8—O1—Li1—O5	−109.67 (15)		

Symmetry codes: (i) −*x*, −*y*, −*z*+2.
